# Therapeutic manipulation of innate lymphoid cells

**DOI:** 10.1172/jci.insight.146006

**Published:** 2021-03-22

**Authors:** Laura M. Cobb, Michael R. Verneris

**Affiliations:** University of Colorado and Children’s Hospital of Colorado, Department of Pediatrics, Center for Cancer and Blood Disorders, Aurora, Colorado, USA.

## Abstract

Since their relatively recent discovery, innate lymphoid cells (ILCs) have been shown to be tissue-resident lymphocytes that are critical mediators of tissue homeostasis, regeneration, and pathogen response. However, ILC dysregulation contributes to a diverse spectrum of human diseases, spanning virtually every organ system. ILCs rapidly respond to environmental cues by altering their own phenotype and function as well as influencing the behavior of other local tissue-resident cells. With a growing understanding of ILC biology, investigators continue to elucidate mechanisms that expand our ability to phenotype, isolate, target, and expand ILCs ex vivo. With mounting preclinical data and clinical correlates, the role of ILCs in both disease pathogenesis and resolution is evident, justifying ILC manipulation for clinical benefit. This Review will highlight areas of ongoing translational research and critical questions for future study that will enable us to harness the full therapeutic potential of these captivating cells.

## Introduction

Innate lymphoid cells (ILCs) are a heterogenous group of cells composed of NK cells and noncytotoxic ILCs that are subcategorized based on transcription factor expression and effector cytokine production (1). ILCs are similar to their Th cell counterparts in terms of functions (2); however, they lack receptors required for antigen specificity (1) (see Table 1 for an overview).

Unlike T cells, ILCs rapidly respond to diverse environmental signals, including alarmins, cytokines, neuropeptides, hormones, and eicosanoids (1). ILCs are largely tissue resident, with some tissue-specific variation seen (3). ILCs have the capacity for plasticity, enabling transdifferentiation into other ILC subtypes according to local environmental cues. ILCs contribute to tissue homeostasis via regulation of tissue metabolism, regeneration, and growth (3). Because of their involvement in key physiologic processes, ILC dysregulation can have devastating consequences, culminating in a broad spectrum of disease. Given their recent identification, our ability to manipulate ILCs for clinical benefit is only now being considered. Nonetheless, recent research demonstrates that therapeutic targeting of ILCs can be achieved via a variety of mechanisms, as discussed below and summarized in Table 2. This Review will highlight conditions in which ILCs are either already targetable or are likely to be successfully targeted based on preclinical studies. ILCs are implicated in more disorders than discussed here, including cancer, which warrants review of its own, as has been done elsewhere (4–6).

## Ex vivo generation of ILCs

Isolation and ex vivo expansion of mature ILCs for the purpose of adoptive transfer has been done successfully in several murine studies. Given the relative paucity of ILCs in peripheral blood, isolation and expansion of ILCs from patients or healthy donors for use in human studies is less feasible. However, as demonstrated by several groups, including our own, human ILCs can be successfully differentiated from umbilical cord blood–derived (UCB-derived) CD34^+^ hematopoietic stem cells (7–12), which could provide a readily available “off-the-shelf” ILC supply for human adoptive transfer studies. Briefly, CD34^+^ cells are cultured on stroma in the presence of various cytokines to generate varying functional ILC subtypes. Using both in vitro and in vivo developmental systems, we have demonstrated that committed human innate lymphoid progenitors (Lin^–^CD34^+^α4/β7^+^ cells) develop into cells that can be distinguished by CD48 and CD52. Lin^–^CD34^+^α4/β7^+^CD48^–^CD52^+^ cells give rise to NK cells, while Lin^–^CD34^+^α4/β7^+^CD48^+^CD52^–^ cells give rise to lymphoid tissue inducer ILC3 cells, and Lin^–^CD34^+^α4/β7^+^CD48^–^CD52^+^ cells give rise to ILC1s, ILC2s, and ILC3s (13). Modulation of both cytokines and surface receptor signaling thus directs development of differing ILC subtypes. For instance, CD48-2B4 (CD244) interactions represent a key development choice between ILC2 and NK cell fates (13). As well, given their functional role in gastrointestinal (GI) homeostasis, we have focused on ILC3 cells, showing that ligation of death receptor 3 (DR3) on ILC3s by tumor-like antigen-1 (TL1A) costimulates in vitro–derived ILC3s and further drives expansion (14).

## Airway

Asthma is a heterogenous disease, with a subset of patients demonstrating enhanced type 2 (type 2–high) airway inflammation (15). ILC2s are disease promoting in type 2–high asthma due to direct activation by TSLP, IL-33, and IL-25 in response to allergens, viruses, and environmental stress. Alternatively, ILC2s can be attracted and activated by mast cell–derived lipid mediators, such as PGD_2_ and CysLTs, produced via IgE-dependent antigen responses (16). Compared with patients with mild disease, those with severe asthma have higher numbers of sputum (17, 18) and circulating (17, 19) ILC2s, which are the principal source of type 2 cytokines (17, 19). Following allergen challenge, lung ILC2s increase, while circulating ILC2s decrease in patients with asthma (20, 21). Elevations in lung mast cell–derived PGD_2_ correlate with decreased circulating ILC2s. The receptor for PGD_2_, CRTH2 (the chemoattractant receptor homologous molecule expressed on Th2 cells), on ILC2s thus mediates PGD_2_-mediated trafficking of ILC2s (20).

Short- and long-acting β_2_-adrenergic receptor (β_2_AR) agonists are a mainstay of asthma management as bronchodilators, but they are also likely effective in asthma due to their negative regulation of ILC2s. The β_2_AR-encoding gene (*ADRB2*) is expressed in both murine and human ILC2s. In mice, treatment with a β_2_AR agonist led to decreased ILC2 frequency and cytokine production following IL-33 or intranasal allergen administration. Signaling via β_2_AR stimulation was also shown to inhibit ILC2 proliferation and effector function in a CD4^+^ T cell–independent manner (22).

**Figure 1:**
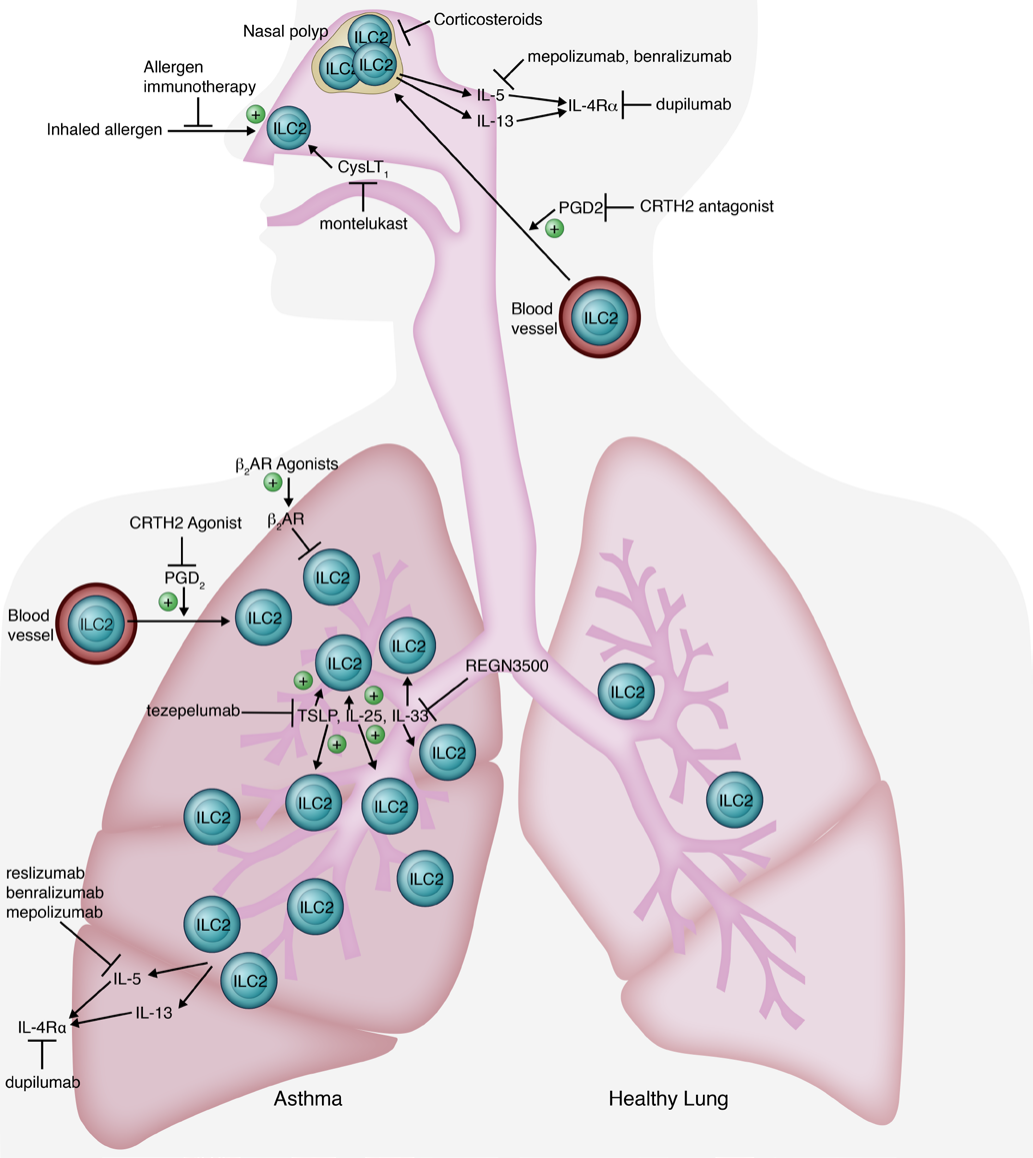
Targeting ILCs in diseases of the airway. Activated ILC2s accumulate in the lung in asthma and promote disease via 2 mechanisms: (a) direct activation by TSLP (thymic stromal lymphopoietin), IL-33, and IL-25 in response to allergens, viruses, and environmental stress and (b) IgE-dependent antigen responses, which induce mast cell secretion of lipid mediators, such as PGD_2_ (prostaglandin D_2_) and CysLTs (cysteinyl leukotrienes), to attract and activate ILC2s. ILC2s also promote type 2 responses in allergic rhinitis (AR) and chronic rhinosinusitis with nasal polyposis (CRSwNP), where they accumulate in polyps. Seasonal increases in ILC2s in AR are suppressed by allergen immunotherapy. Montelukast (a CysLT_1_ antagonist), approved for asthma and AR, inhibits ILC2 activation. β_2_ Adrenergic receptor (β_2_AR) agonists negatively regulate ILC2s in asthma by inhibiting their proliferation and effector function. In both asthma with or without AR and CRSwNP, systemic steroids decrease ILC2 cytokine production and promote ILC2 apoptosis. Dupilumab inhibits IL-4Rα, the shared receptor of IL-4 and IL-13, and is approved in asthma and CRSwNP. Anti–IL-5 mAbs (mepolizumab, benralizumab, reslizumab) are approved in asthma and are being tested in CRSwNP. CRTH2 (chemoattractant receptor-homologous molecule expressed on Th2 cells) antagonists to block PGD_2_-mediated ILC2 trafficking as well as antibodies against TSLP and IL-33 to inhibit ILC2 activation are in testing. Illustrated by Rachel Davidowitz.

Several mAbs targeting ILC2 effector cytokines have received FDA approval for asthma treatment. As illustrated in Figure 1, mepolizumab, benralizumab, and reslizumab all target IL-5, while dupilumab inhibits IL-4Rα, the shared receptor of IL-4 and IL-13. The anti–IL-33 mAb, REGN3500, has completed phase II testing (15), and phase III trials for tezepelumab, an anti-TSLP ILC2 activation-inhibiting mAb, are underway (23) following promising phase II results (24).

As in type 2–high asthma, ILC2s promote type 2 responses in allergic rhinitis (AR). Circulating ILC2s are significantly increased in AR (19, 25) and correlate with symptom severity and plasma IL-13 levels (25). Interestingly, seasonal increases in ILC2s are suppressed by allergen immunotherapy (26). Additionally, montelukast (CysLT_1_ antagonist), approved for asthma and AR (27), is likely efficacious in part by inhibiting ILC2 activation, as montelukast blocked mast cell production of ILC2-activating cytokines in vitro (28).

Chronic rhinosinusitis with nasal polyposis (CRSwNP) is another type 2–driven condition. ILC2s are significantly elevated in nasal polyps (29, 30) and spontaneously secrete IL-5 and IL-13 (29). Dupilumab (anti–IL-4Rα) is approved for CRSwNP (31), while IL-5–targeting mAbs benralizumab and mepolizumab are expected to receive FDA approval following positive phase III results (32, 33). CRTH2 antagonists, which are expected to block PGD_2_-mediated ILC2 trafficking, are being developed for both CRSwNP and asthma. The CRTH2 antagonist GB001 showed promising phase II results for asthma (34), while both GB001 and ACT-774312 have completed phase II testing for CRSwNP and are awaiting results (35, 36).

Finally, in both asthma (with or without AR) and CRSwNP treatment with systemic steroids reduces ILC2s (18, 19, 30). Steroids also dramatically decrease ILC2 cytokine production by inhibiting STAT signaling (19) and promoting ILC2 apoptosis (30). In asthma, a steroid-mediated decrease in ILC2s improved symptoms, despite preserved Th17 T cells and eosinophils (18), supporting the role of ILC2s in disease pathogenesis.

## Blood and marrow transplantation

ILCs can be both protective and therapeutic in acute graft-versus-host disease (aGVHD), a frequent, often life-threatening complication of allogeneic stem cell transplant (alloSCT). ILCs demonstrate variable survival and repopulation capacity following chemotherapy and/or irradiation (37). A natural cytotoxicity receptor–negative (NCR^–^) ILC3 population in the intestine and thymus are radioresistant. After transplant, these recipient-derived, IL-23–responsive ILC3s produce IL-22, which acts on thymic epithelial cells and gut stem cells, facilitating donor T cell recovery and preventing aGVHD (37, 38). Further, GVHD depletes these tissue-protective ILCs, eliminating the critical source of IL-22 that acts to maintain intestinal stem cells and the epithelial barrier itself (37). In contrast, intestinal ILC2s are sensitive to conditioning and limited in their ability to repopulate from donor BM. Strikingly, adoptive transfer of IL-33–activated ILC2s reduced GI aGVHD and improved survival in established murine aGVHD. ILC2s suppress alloreactive T cell production of IFN-γ and IL-17A via IL-13–dependent recruitment of myeloid-derived suppressor cells. Moreover, ILC2 infusion improved GI tract mucosal integrity without impairing graft-versus-leukemia response, as had been seen with Treg infusion (39).

Patients undergoing alloSCT showed an association between circulating ILCs and GVHD susceptibility. Patients with increased circulating activated CD69^+^ ILC2s and NCR^+^ ILC3s before alloHSCT did not ultimately develop gut GVHD. Furthermore, skin-homing NCR^–^ ILC3s and ILC1s were increased in patients without skin aGVHD compared with those with skin aGVHD. These results suggest that ILCs could offer protection against alloHSCT-associated tissue damage, thereby decreasing subsequent GVHD risk (40).

ILCs may also exert direct immunosuppressive effects to protect against GVHD. A novel subset of ILC3s localized to the GI tract and BM was recently described (41). These Ecto^+^ ILC3s (largely NCR^+^) coexpress ectoenzymes, CD39 and CD73, capable of hydrolyzing NAD^+^ and extracellular ATP (eATP). eATP-induced Ecto^+^ ILC3s suppress T cell proliferation by producing adenosine and promoting tissue repair via IL-22. Intriguingly, alloHSCT patients with gut GVHD had significantly fewer gut Ecto^+^ ILC3s and decreased serum adenosine and its metabolite inosine. Following tissue injury, damage-associated molecular patterns (DAMPs), including ATP, are released. Ecto^+^ ILC3s might mitigate antigen-presenting cell activation via adenosine production, while also producing IL-22 that promotes tissue repair, thereby maintaining gut integrity and mitigating GVHD (41). Collectively, these results support further investigation on the use of expanded ILC2s and/or NCR^+^ ILC3s as a cellular therapy to both prevent and treat aGVHD in patients undergoing alloHSCT.

## Cardiology

ILC1s and ILC2s play opposing roles in atherosclerosis pathogenesis. Increased circulating ILC1s are present in patients with acute ST-segment elevation myocardial infarction (42) and atherosclerotic cerebral infarction, where they correlate with higher oxidized LDL levels (43), suggesting ILC1s are proatherogenic. In contrast, atheroprotective ILC2s are decreased in patients with myocardial (42) or cerebral (43) infarcts and in high-fat diet–fed mice (44). Moreover, genetic ILC2 ablation promotes atherosclerosis development (44), while ILC2 expansion via administration of IL-2 (45), IL-25 (46), or adoptive transfer is protective (47). Mechanistically, increased ILC2s (and therefore IL-5 secretion) expand B1a cells and production of anti-phosphorylcholine antibodies targeting oxidized LDL (46), while IL-13 induces alternatively activated macrophages (AAMs) important for tissue repair (44). A clinical trial to evaluate low-dose IL-2, including effects on ILC2s, in acute coronary syndrome is planned (48).

ILCs are increased in the pericardial fluid of patients with pericarditis. An IL-33–induced pericarditis model demonstrates that ILC2s are indispensable in the development of eosinophilic pericarditis. Blocking IL-5 was protective, suggesting that therapeutically targeting the IL-33/ST2/ILC2s axis might be a viable treatment strategy for eosinophilic pericarditis (49).

## Dermatology

ILCs are implicated in psoriasis, as NCR^+^ ILC3s are increased in the skin (50, 51) and blood (50–52) of patients with psoriasis. Skin NCR^–^ ILC3s stimulated with IL-23 convert to NCR^+^ IL-22–producing ILC3s that contribute to epidermal thickening characteristic of disease (50). Recent work also shows that ILC2s can transdifferentiate into NCR^–^ ILC3s and produce IL-17 in response to IL-1β and IL-23 (53). Patients with active disease have decreased circulating and skin ILC2s (51, 53), probably due to transdifferentiation into ILC3s. Both NCR^–^ and NCR^+^ ILC3s express skin-homing receptors (51), and in active psoriasis, circulating NCR^–^ ILC3s are decreased (51), likely due to NCR^–^ ILC3 CCR10-mediated homing to skin. Interestingly, successful treatment with TNF-α inhibition decreases circulating NCR^+^ ILC3s, while increasing NCR^–^ ILC3s (51). The rise in circulating NCR^–^ ILC3s seen with disease improvement (51) likely reflects extravasation of these cells from previously active skin lesions. Given that TNF-α synergizes with IL-23 to promote IL-17A production by ILCs (54), it is likely that TNF-α and IL-23 inhibition are effective for psoriasis at least in part due to their ILC3 suppressive effects, thereby reducing IL-17A. Further, several additional agents directly targeting IL-17A or its receptor have all been approved for use in psoriasis (55).

Activated ILC2s are increased in atopic dermatitis (AD) skin lesions and targeting their effector function ameliorates disease. Dupilumab (anti–IL-4Rα), which inhibits the shared IL-4 and IL-13 receptor, is approved for AD (31); tralokinumab (anti–IL-13) has been submitted for approval (56); and lebrikizumab (anti–IL-13) is currently in phase III trials for AD (57, 58).

Additionally, numerous JAK inhibitors are in various phases of clinical testing for both psoriasis (55) and AD (59). JAK inhibitors modify cytokine receptor signaling and thus regulate ILC effector cytokine production and/or affect ILC plasticity. IL-4, IL-13, and IL-5 signaling via JAK-STAT can be therapeutically restrained in AD, while inhibition of IL-23 signaling in psoriasis can prevent transdifferentiation into pathogenic ILC subtypes (50, 53, 55).

ILC2s are also critical in reepithelialization of cutaneous wounds. ILC2s accumulate at the site of injury and persist in elevated numbers 5 days after wound induction. IL-33–deficient mice have significantly less accrual of activated ILC2s at sites of injury, corresponding to significantly delayed wound closure compared with WT mice. Additionally, ILC2-depleted mice experience significant impairment of reepithelialization. Treatment with recombinant IL-33, however, significantly increases reepithelialization at 5 days after wounding, likely due at least in part by supporting induction of AAMs (60). Therefore, exogenous IL-33 and/or adoptive transfer of ILC2s might be of clinical utility in accelerating wound healing.

## Endocrinology

**Figure 2:**
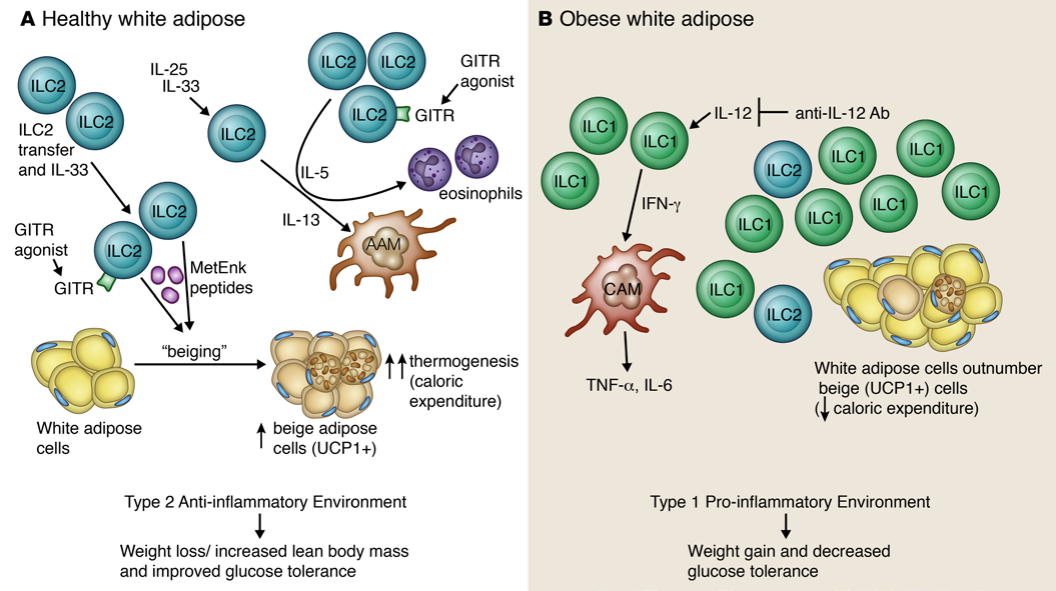
Targeting ILCs in obesity and type 2 diabetes mellitus. In healthy white adipose (**A**), ILC2s promote an antiinflammatory type 2 environment via eosinophil recruitment and induction of alternatively activated macrophages (AAMs). Specialized beige adipocytes interspersed in WAT can utilize the mitochondrial uncoupling protein 1 (UCP1) for thermogenesis, increasing caloric expenditure and protecting from obesity. “Beiging” is promoted by ILC2 production of methionine-enkephalin (MetEnk) peptides. Thus, expansion of ILC2s via IL-25, IL-33, or ILC2 adoptive transfer promotes weight loss and improved glucose tolerance. Additionally, engagement of the glucocorticoid-induced TNF receptor (GITR) on ILC2s via a GITR agonist also promotes beiging. ILC2s are decreased in obese adipose (**B**), while ILC1s are increased. IL-12–dependent IFN-γ production is sustained by adipose ILC1s and induces classically activated macrophages (CAMs) to secrete IL-6 and TNF-α. This promotes a proinflammatory type 1 environment, which fosters development of glucose intolerance and leads to type 2 diabetes mellitus. Therefore, therapeutically activating the IL-33/GITR/ILC2-beiging pathway signifies a novel method for treating obesity and T2DM. Illustrated by Rachel Davidowitz.

A distinct subset of adipose-resident non-NK ILC1s is implicated in obesity and metabolic dysfunction, including type 2 diabetes mellitus (T2DM). Mice fed a high-fat diet exhibit selective proliferation and accumulation of ILC1s within subcutaneous adipose (61). Adoptive transfer of adipose ILC1s from high-fat diet–fed mice exaggerated ILC1 accumulation in recipients and amplified glucose intolerance (62). As illustrated in Figure 2, early in obesity, IL-12–dependent IFN-γ production is sustained locally in adipose, most prominently by ILC1s. This ILC1-mediated IFN-γ production potently induces classically activated macrophages to promote a dysregulated proinflammatory environment, fostering insulin resistance (61). Moreover, coculture of BM-derived macrophages with adipose ILC1s increased macrophage proinflammatory cytokines (IL-6 and TNF-α), an effect abolished by anti–IFN-γ. Likewise, adoptive transfer of IFN–γ-deficient adipose ILC1s did not cause the glycemic intolerance seen with transfer of WT adipose ILC1s. Notably, anti–IL-12 decreased ILC1 and CD11c^+^ macrophage accumulation and reduced glucose intolerance, adipose fibrosis, hepatic steatosis, and serum-free fatty acid levels. As further validation, ILC1s are increased in the adipose of obese patients and even higher in obese patients with T2DM. Adipose ILC1s from obese patients demonstrate higher IFN-γ expression than healthy controls. Moreover, circulating and adipose ILC numbers positively associate with blood glucose levels and hemoglobin A1c. Interestingly, circulating ILC1s are significantly diminished after bariatric surgery and correlate with decreased BMI and improved glycemic parameters (62). This exciting work confirmed adipose ILC1s as a therapeutic target given their critical pathophysiologic role in metabolic syndrome.

As shown in Figure 2, ILC2s also reside in white adipose tissue (WAT), where they are decreased in obesity (63, 64). Stimulating ILC2s via administration of IL-25 (65) or IL-33 (63) promotes an antiinflammatory type 2 environment via eosinophil recruitment and AAM induction, while ILC2 depletion causes defective accumulation of these cell types (63, 65). Treatment of obese mice with IL-25 (65), IL-33 (64), or adoptive ILC2 transfer (64, 65) increased adipose ILC2s, resulting in weight loss and improved glucose tolerance. In contrast, ILC2 depletion exaggerated obesity and impaired glucose tolerance on a high-fat diet (65). Further, mice deficient in IL-33, IL-13 (65), or IL-5 (63) all demonstrated exacerbated weight gain and impaired glucose homeostasis.

Unlike WAT, brown adipose utilizes mitochondrial uncoupling protein 1 (UCP1) for thermogenesis to generate body heat and protect from obesity. Recent work demonstrated the existence of specialized beige adipocytes interspersed within WAT. Unlike typical white adipocytes, beige cells induce uncoupling-dependent thermogenesis to expend calories as robustly as classical brown adipocytes (66). Mice treated with IL-33 or ILC2 adoptive transfer had increased numbers of UCP1^+^ beige cells within WAT, a process termed “beiging.” IL-33–mediated effects were ILC2 dependent but occurred independently of eosinophils, IL-4 receptor signaling, and adaptive immune cells. Furthermore, ILC2s can produce methionine-enkephalin (MetEnk) peptides, which upregulate UCP1 in adipocytes in vitro and promote WAT beiging in vivo, which can be enhanced by IL-33 (64).

Both human and murine adipose ILC2s also express GITR. GITR agonists activate ILC2 effector function, identifying GITR as an immune costimulatory checkpoint for ILC2s. GITR engagement on activated ILC2s not only stimulates secretion of ILC2 effector cytokines but also prevents ILC2 apoptosis. Strikingly, a GITR agonist protected against murine T2DM and reversed established glucose intolerance by inducing adipocyte beiging. These effects were contingent on ILC2-derived cytokine secretion, especially IL-13 (67). Thus, therapeutic activation of the IL-33/GITR/ILC2/beiging pathway represents a novel method for treating obesity and associated metabolic disorders, including T2DM.

## Gastroenterology

ILCs have received attention for their role in Crohn’s disease (CD), a subtype of inflammatory bowel disease (IBD). Environmental signals, including those from myeloid cells (68), regulate ILC frequency and their function in the gut. Proinflammatory IL-12 produced by DCs stimulates ILC1s to produce IFN-γ and TNF-α (69). In CD, ILC1s accumulate and become the predominant subset in the inflamed intestine (70–72). This ILC1 accumulation occurs at the expense of NCR^–^ ILC3s, which are reduced and correlate with disease severity (72). mRNA analysis of ileal tissue from patients with CD revealed marked upregulation of *IFNG*, *TNFA,* and *TBET* transcripts (71). IL-12 not only stimulates ILC1s, but also induces transdifferentiation of NCR^–^ and NCR^+^ ILC3s into ILC1s, likely accounting for the observed alteration in ILC1/ILC3 ratio (70).

ILC1 plasticity also permits differentiation into IL-22–producing NCR^+^ ILC3s under the influence of IL-23 (70). While ILC3s maintain gut homeostasis (3), they can also become dysregulated and contribute to intestinal inflammation. ILC3s are indispensable to the development of bacterial-driven colitis, where they produce IL-17 and IFN-γ in response to IL-23 (73). ILC3s are also implicated in an IL-23/GM-CSF–mediated autocrine feedback loop. Early in the inflammatory response, IL-23 secretion by myeloid cells is GM-CSF dependent, and GM-CSF production by ILC3s is sustained by IL-23. Indeed, increased numbers of GM-CSF– and TNF-α–producing ILC3s are present in the blood of patients with CD, and GM-CSF^+^ ILC3s are further enriched in the colon. GM-CSF not only recruits myeloid cells, but it may also mobilize ILC3s. In homeostatic states, ILC3s reside largely within lymphoid aggregate cryptopatches (CPs) rather than the intestinal mucosa. Following colitis induction, ILC3s exit CPs and migrate into adjacent intestinal mucosa. GM-CSF mAb treatment prevents ILC3 egress from CPs, thus indicating local ILC3 migration is GM-CSF–dependent (74).

**Figure 3:**
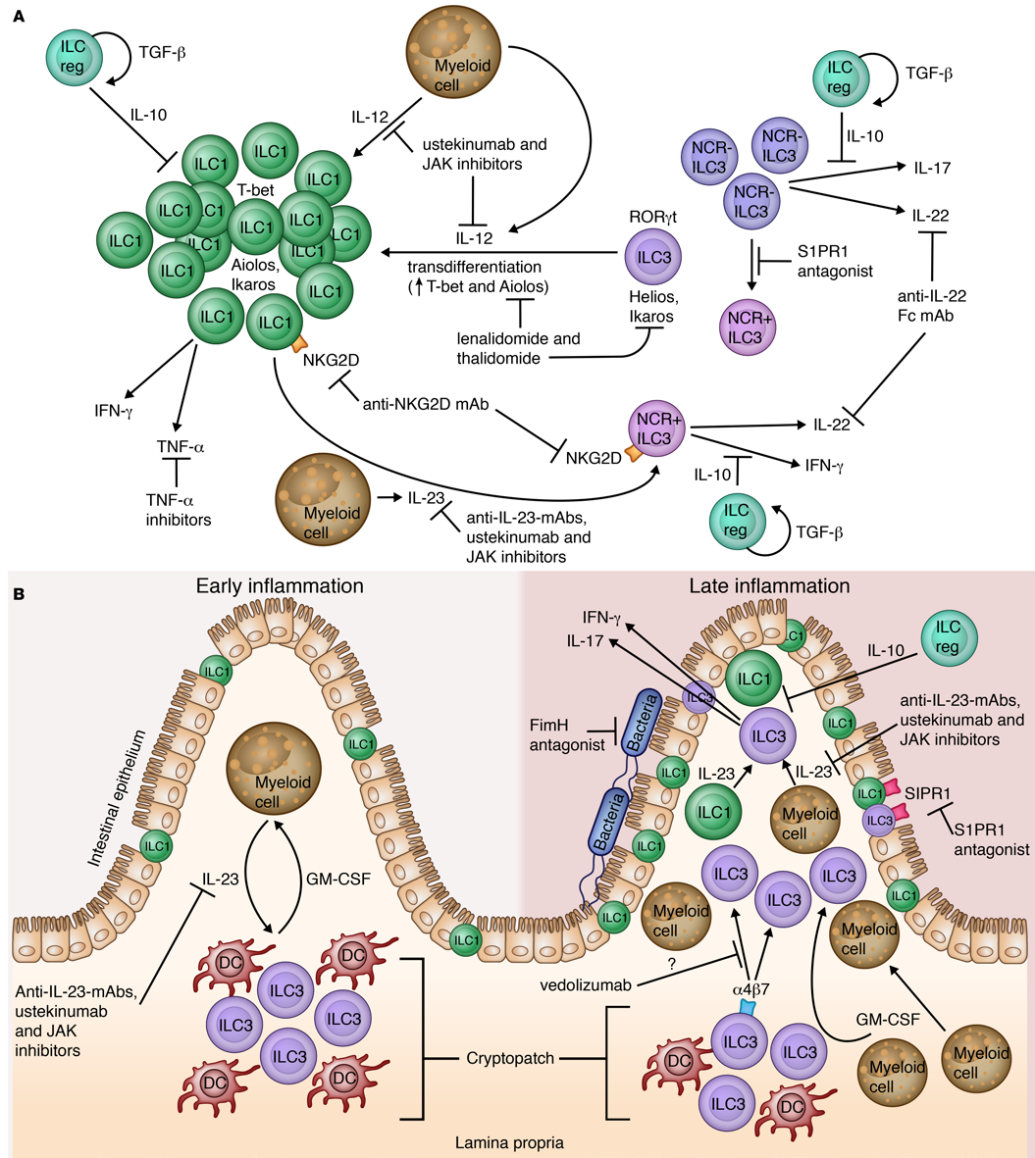
Targeting ILCs in Crohn’s disease. (**A**) In Crohn’s disease (CD), ILC1s accumulate in the intestine, while NCR^–^ ILC3s are reduced. Myeloid cells, including DCs, produce IL-12, stimulating ILC1s to produce IFN-γ and TNF-α. IL-12 also induces transdifferentiation of NCR^–^ and NCR^+^ ILC3s into ILC1s. IL-23 promotes differentiation of ILC1s into IL-22–producing NCR^+^ ILC3s. Regulatory ILCs (ILCregs) downregulate ILC1s and ILC3s via IL-10 secretion to suppress production of cytokines other than IL-22. ILCregs also produce TGF-β, which promotes their own expansion and survival. Thalidomide and lenalidomide modulate ILC transdifferentiation by selectively degrading Aiolos (ILC1 specific) and Ikaros (ILC nonspecific), increasing ILC3-associated Helios. Sphingosine-1 phosphate receptor 1 (S1PR1) antagonists downregulate ILC3 NCR expression. TNF-α inhibitors directly block TNF-α produced by ILC1s. Ustekinumab and JAK inhibitors target IL-12 and IL-23, while IL-23–specific mAbs target IL-23 and an anti–IL-22 Fc mAb targets IL-22. An anti-NKG2D mAb might modulate ILC1s and NCR^+^ ILC3s, which express NKG2D in mice. (**B**) In healthy intestine, ILC3s reside largely within lymphoid aggregate cryptopatches (CPs). Early in inflammation (left), IL-23 secretion by myeloid cells is GM-CSF dependent, and GM-CSF production by ILC3s is sustained by IL-23. Later in inflammation (right), GM-CSF recruits additional myeloid cells and mobilizes ILC3s from CPs into adjacent intestinal mucosa. In response to IL-23, ILC3s produce IL-17 and IFN-γ, promoting intestinal inflammation. IL-23 can be blocked by anti–IL-23 mAbs, ustekinumab, and JAK inhibitors. α4β7 Integrin blockade with vedolizumab might inhibit ILC migration from CPs into intestinal mucosa. A FimH antagonist reduces bacterial adherence to the gut, decreasing innate immune activation and improving intestinal permeability. S1PR1 antagonists prevent ILC1/3 maturation and migration and modify ILC functions. Illustrated by Rachel Davidowitz.

Several ILC-targeting biologic therapies are already approved for CD (shown in Figure 3). TNF-α inhibition is efficacious in many patients. The α4β7 integrin inhibitor vedolizumab and IL-12/23 p40 mAb ustekinumab are also approved for CD (75). There is evidence that biologic efficacy in CD is mediated at least partially by modulating ILC differentiation, function, and/or migration, thereby promoting normalization of ILC frequencies in the intestine. In 54 patients with IBD (including 5 patients with ulcerative colitis), intestinal ILC frequencies were assessed before initiation of TNF-α inhibitor, ustekinumab, or vedolizumab. At baseline, intestinal NCR^+^ ILC3s were notably decreased while ILC1s were increased in nearly all patients. Strikingly, all treatments led to significant NCR^+^ ILC3 recovery, regardless of biologic type. Patients treated with TNF-α blockade and ustekinumab also showed increases in circulating NCR^+^ ILC3s (76), which were not observed with vedolizumab (72, 76). Interestingly, the α4β7 integrin blocked by vedolizumab is known to be critical for ILC development and migration (13, 77). However, the lack of change in circulating ILCs with vedolizumab suggests it is unlikely to prevent homing of ILCs from the blood to the gut (72). Rather, vedolizumab might inhibit ILC migration from CPs into intestinal mucosa. Additional published findings support biologic-mediated ILC modulation leading to clinical efficacy in CD. A patient with CD with high levels of intestinal ILC1s and decreased ILC3s before treatment had decreased ILC1s and increased ILC3s following ustekinumab therapy. Ustekinumab also associated with decreased *IL12A*, *IL17A*, *IL22*, and *IL23A* mRNA expression, despite unchanged *IFNG*, *TNFA*, and *TBET* expression. Most importantly, this patient’s shift toward normalization of ILC frequencies as a result of ustekinumab was accompanied by intestinal mucosal healing (71).

Several other therapies are in development that likely target ILCs (Figure 3). Multiple IL-23 p19 mAbs are in phase II or III trials for CD with promising outcomes (78) in addition to a phase II trial of an IL-22 Fc (79). Several JAK inhibitors have also shown efficacy in trials (78) and likely affect ILC plasticity as a result of modifying cytokine receptor signaling, including IL-12 and IL-23. An anti-NKG2D mAb is being tested in a phase II trial (80). Given that NKG2D is expressed on murine intestinal ILC1s and NCR^+^ ILC3s (69), this drug might also modulate identical subtypes in humans. Targeting the gut microbiome may also affect ILCs, given that microbial cues modify ILC phenotype and function (81). A phase II study is underway using Sibofimloc, a small-molecule FimH antagonist designed to reduce bacterial adherence to the gut, thereby decreasing innate immune activation and reducing intestinal permeability (82). S1PR1 is expressed by ILC1s and ILC3s (83), and S1PR1 antagonists are expected to prevent their maturation and migration (84). Mice treated with S1PR1 antagonist fingolimod had a significant reduction in small intestine ILC3s (83). Ex vivo treatment of ILC3s and ILC1s reduced cytokine production and downregulated ILC3 NCR expression in a dose-dependent fashion. Intriguingly, fingolimod did not reduce murine intestinal ILC3 cytokine levels, despite decreased ILC3 numbers. Rather, fingolimod-treated mice had higher IL-22, GM-CSF, and IL-17A relative to vehicle-treated animals. Thus, S1PR1 antagonists likely modify ILC functions, triggering other non-ILC cells to secrete these cytokines, thereby maintaining mucosal barrier immunity (83). In addition to a phase III trial for fingolimod in CD (85), there are several phase II/III studies underway for other S1PR1 antagonists, including ozanimod (86) and etrasimod (87). Small molecules can also modulate ILC transdifferentiation, thanks to recent work uncovering transcription factor regulators of ILC3-ILC1 plasticity. The transcription factor Ikaros is expressed by all ILCs; however, Aiolos is expressed predominantly by ILC1s and Helios is associated with ILC3s. Transdifferentiation of ILC3s into ILC1s involves upregulation of T-bet and Aiolos. Treatment with the small molecule, lenalidomide, a thalidomide analog, caused selective degradation of Aiolos and Ikaros, suppressed ILC1 differentiation, and increased ILC3-associated Helios (88). A phase III study is currently investigating the use of thalidomide in CD, based on previous indications of clinical efficacy (89).

Finally, regulatory ILCs (ILCregs) are a recently proposed type of gut ILC that suppress ILC1 and ILC3 activation by secreting IL-10, thus promoting inflammation resolution. ILCreg depletion promotes ILC1/3 activation, leading to more severe intestinal inflammation, as Tregs have no appreciable inhibitory effect on ILCs. In contrast, adoptive transfer of ILCregs protects mice from colitis by suppressing ILC-mediated cytokine suppression through IL-10. Interestingly, ILCregs inhibit IFN-γ and IL-17A production but not that of IL-22. Further, ILCregs secrete TGF-β in response to inflammation, which promotes their own survival and expansion (90). Of note, the existence of ILCregs as a distinct subset has been called into question following research indicating that ILCregs might actually be gut ILC2s that can be induced to secrete IL-10 (91). Nonetheless, the importance of IL-10 is confirmed by the fact that the *IL10* gene is contained within an identified CD susceptibility locus and *Il10*-KO mice are a highly regarded IBD model (92). Previous trials using IL-10 in CD did not demonstrate obvious clinical improvement (93); however, it is tempting to speculate that local IL-10 production via a microbiome-derived biologic product (82) or infusion of ILCregs themselves might be efficacious.

## Hepatology

Liver ILC1s protect against nonfulminant acute liver injury (ALI) induced by carbon tetrachloride (CCl_4_) or low-dose acetaminophen in mice. In addition, ILC1 deficiency is associated with more severe injury, while adoptive transfer of ILC1s is protective. Liver ILC1-derived IFN-γ acts to restrain hepatic stellate cell (HSC) activation, which otherwise intensifies injury and subsequent fibrosis. Further, IFN-γ selectively upregulates B cell lymphoma–extra large (Bcl-xL) in injured hepatocytes, promoting their survival. Notably, IFN-γ itself has dual roles in liver injury. IFN-γ is injurious at very high concentrations, as seen in fulminant ALI (i.e., following high-dose acetaminophen), but protective at lower concentrations induced by mild injury (i.e., low-dose acetaminophen or bile duct ligation). The mechanisms regulating ILC1-mediated IFN-γ are currently undefined.

ILC1s express a variety of NK cell receptors, the functions of which remain largely unknown. DNAX accessory molecule-1 (DNAM-1), an activating receptor, is preferentially expressed on ILC1s following ALI. IL-7, which regulates hepatic lymphocyte responses, is produced by hepatocytes themselves. Signaling via both DNAM-1 and the IL-7 receptor is required to optimally activate ILC1s following ALI. Tissue injury, such as ALI, induces release of DAMPs, including ATP. Liver ILC1s express the purinergic receptor P2X7 (P2RX7), an ATP-gated cation-selective ion channel that permits Ca^2+^ influx. ATP-mediated P2RX7 signaling is obligatory for optimal ILC1 IFN-γ secretion. ATP additionally enhances ILC1 IFN-γ production induced by DC-derived IL-12. This effect of ATP is perhaps mediated by the increased intracellular calcium concentration in ILC1s that occurs with ATP/P2RX7 signaling (94). While exogenous administration of IFN-γ has therapeutic appeal for mild ALI, the risks of such therapy are obvious. This research does, however, highlight the need for further investigation into the functions of NK cell receptors on ILC1s. Such work could provide insight into methods to modulate ILC1 function to prevent tissue damage and subsequent organ failure.

In contrast, ILC2s mediate liver fibrosis, and their increased frequency directly correlates with disease severity (95). Chronic hepatocyte stress triggers IL-33 release, promoting ILC2 accumulation and activation. ILC2 secretion of IL-13 then acts via IL-4Rα and STAT6-dependent signaling to promote HSC activation and transdifferentiation into myofibroblasts. The critical role of IL-33 was shown using IL-33–deficient mice, which develop significantly less fibrosis than WT mice (96). Similarly, IL-33–induced ILC2 expansion fosters disease progression in immune-mediated hepatitis (97). In both hepatic fibrosis and immune-mediated hepatitis, ILC2 depletion effectively ameliorates disease, while ILC2 adoptive transfer exacerbates disease severity (96, 97). Thus, therapies manipulating IL-33–ILC2 responses could prove valuable in treating hepatic inflammatory disease and fibrosis.

## Nephrology

An initial study evaluating therapeutic IL-25 was undertaken in a model of chronic kidney disease (CKD), in which doxorubicin administration causes focal segmental glomerular sclerosis-like disease. In this model, IL-25 reduced glomerular sclerosis, tubular atrophy, interstitial expansion (fibrosis), and proteinuria. IL-25 administration also increased IL-4, IL-5, IL-13, and AAMs, effects that were abrogated by IL-4/13-neutralizing antibodies (98). While ILCs were not investigated in this initial study, a subsequent study found that ILC2s were the predominant subtype in both mouse and human kidneys. Short-term, low-dose IL-33 caused prolonged ILC2 expansion and protected against CKD. IL-33 also increased IL-5 and IL-13 along with marked eosinophil expansion, decreased neutrophil infiltration, and AAM induction. Of note, eosinophils were obligatory for IL-33–mediated effects. Furthermore, IL-33 was protective in T cell–deficient but not in ILC-deficient mice, supporting ILCs in renoprotection (99). More recently, a combination of IL-33 and IL-2 (as IL233) was also protective against CKD, likely due to expansion of both ILC2s and Tregs (100, 101). Remarkably, IL233 also increased expression of multiple renal progenitor cell markers, indicating it may also promote an environment conducive to regeneration (101).

Ischemia/reperfusion injury (IRI) is the foremost cause of acute kidney injury (AKI). IRI effects include tubular cell vacuolization, dilation, and necrosis with cast formation (102). In IRI, ILC2s are reduced and ILC1 and ILC3s increased (103). Treatment with low-dose IL-25, IL-33, or IL233 before IRI induced ILC2 expansion and preserved renal function, reduced neutrophil infiltration, and tubular injury. Furthermore, ILC2 adoptive transfer before IRI was also protective (100, 102, 104). Comparable efficacy was seen in a humanized mouse model following both IL-33 and adoptive transfer of human ILC2s. Moreover, the beneficial effects of low-dose IL-33 were unaffected by selective Treg depletion and partially reduced by macrophage depletion but completely eliminated when ILC2s were absent, confirming a critical role of ILC2s in IRI-mediated renoprotection (104).

As seen in CKD, administration of IL-25 or IL-33 before IRI was accompanied by type 2 cytokine production and AAM induction (102, 104). IL-4/13–neutralizing antibodies blocked AAM induction in both CKD and IRI (98, 102). Notably, ex vivo–stimulated ILC2s increase amphiregulin (Areg) production and selective *Areg* deletion in ILC2s abolishes protective effects in IRI (104). In contrast, Areg was not increased following in vivo IL-33 treatment in CKD (99). These differential effects on Areg production suggest that ILC2s employ different mechanisms of renoprotection in an environment and/or disease-specific context. Differences in ILC2-mediated effects might also depend on the involvement of cooperative cell types, such as Tregs. This could explain why IL-33, which modestly increases Tregs, depends on type 2–mediated eosinophil expansion in CKD (99), but IL233, which expands both ILC2s and Tregs, does significantly upregulate type 2 responses in IRI (100). Studies investigating IL-33 in renal injury also highlight a less-is-more strategy when it comes to therapeutic benefit. IL-33, an alarmin, is upregulated with tissue injury, including injury induced by either doxorubicin (101) or cisplatin (105). Prolonged IL-33 treatment worsens fibrosis following IRI (106). Further, short-duration but high-dose IL-33 exacerbated AKI in a cisplatin-induced model (105). These studies directly contrast the beneficial effects described for short-term, low-dose IL-33 administration in which exogenous IL-33 lowers renal IL-33 levels (101).

ILCregs have also been therapeutically targeted to treat renal IRI. Interestingly, ILCregs from mice subjected to IRI generated less IL-10, indicating a loss of endogenous suppressive function. When expanded ex vivo, ILCregs increased IL-10 and TGF-β production, inhibiting proinflammatory cytokine production by both ILC1s and classically activated macrophages. Treatment with IL-2C (IL-2/anti–IL-2 Ab complex) markedly increased CD25 expression on ILCregs, leading to ILCreg expansion in T cell–deficient mice and proliferation of both ILCregs and Tregs in WT mice. IL-2C–mediated renoprotection was eliminated following ILCreg depletion. Furthermore, adoptive transfer of ILCregs mitigated renal injury. Treatment with IL-2C or ILCreg infusion diminished neutrophil infiltration, induced AAMs, and decreased proinflammatory ILC1s (107).

## Neurology

Murine multiple sclerosis (MS) models show that ILCs can drive neuroinflammation. T-bet–dependent NCR^+^ ILCs mediate Th17 accumulation in the CNS (108). Patients with MS have elevated ILC3s in both blood (109) and cerebrospinal fluid (110). Several S1PR1 antagonists, including fingolimod, siponimod, and ozanimod, are already approved for use in MS (111). Patients with MS on fingolimod have dramatically reduced total circulating ILCs, with reductions of all ILC subsets (83), suggesting that S1PR1 modulators are efficacious in MS by inhibiting ILC (particularly ILC3) migration to CNS, thereby limiting Th17 infiltration.

Alternatively, ILC2s are beneficial in neurologic injury and aging. Lung-derived ILC2s adoptively transferred into the subarachnoid space of mice notably improve functional recovery following spinal cord injury (112). A more recent study found that ILC2s accumulate in the choroid plexus with aging and can limit aging-associated neuroinflammation via IL-5 production. Enhancing IL-5 by increasing ILC2s, either through IL-33 or adoptive ILC2 transfer, markedly improves cognitive function of aged mice (113).

## Solid organ transplantation

NCR^+^ ILC3s can defend against hepatic IRI, a significant cause of liver transplant graft failure. NCR^+^ ILC3-deficient mice develop severe hepatic IRI that is reversed with adoptive transfer of NCR^+^ ILC3s. Adoptive transfer of IL-22–deficient cells abolished beneficial effects, confirming hepatoprotection is IL-22 dependent. Excitingly, IL-22–producing NCR^+^ ILC3s were successfully isolated from ex vivo–perfused livers, suggesting the possibility of using donor liver perfusates as a cellular source of NCR^+^ ILC3s for adoptive transfer into liver transplant recipients to prevent hepatic IRI (114). Similarly, IL-22–ILC3–directed interventions might also improve outcomes in other types of solid organ transplant, including lung (115), intestine (116), and pancreatic islet (117) transplantation, where IL-22 production mediates graft tolerance.

## Concluding remarks

Substantial progress has been made in understanding ILC development and biology; however, ILCs continue to represent an enigmatic population with seemingly boundless clinical implications. There are several challenges that currently limit our grasp on ILC function in disease, thereby constraining the ability to manipulate these cells therapeutically. Growing evidence from murine studies confirms the existence of a distinct ILC1 population, allowing for study of ILC1-mediated roles in disease models. Conversely, no markers have been conclusively identified to distinguish human ILC1s (118), highlighting a critically important area to address for understanding the role of ILC1s in human disease. Further, the exceptionally adaptable functions and plasticity of ILCs are complicated by the fact that ILC functions can differ based on tissue (119). For example, a recent study found that ILC2s isolated from the small intestine actually induce obesity on adoptive transfer (120), a result in stark contrast to the protection provided by adipose-derived ILC2s. Despite the often complex pathophysiologic mechanisms involved, it is clear that ILCs can play opposing roles, both driving disease and promoting its resolution. Here, we have attempted to highlight those disease processes in which ILCs are already targeted or could feasibly be targeted for therapeutic benefit. The growing myriad of mAbs and small molecules that have been developed make direct or indirect targeting of ILC effector functions, in vivo expansion, transdifferentiation, modulation, and even migration a feasible approach. Further, the ability to reliably differentiate human ILC subtypes from cord blood stem cells provides a viable method for using adoptively transferred ILCs as a form of cellular therapy. However, when evaluating therapeutic targeting of ILCs we must carefully consider that ILC functions cannot be reliably inferred by their phenotype but rather depend on a combination of ontogeny and local environmental influences (119). Further, the effects of modulating one ILC subtype might have unforeseen, possibly detrimental effects on other ILCs, immune cells, or tissues. Thus, additional studies are needed to further elucidate mechanisms of ILC plasticity, delineate the regulatory mechanisms that influence ILC functions, and examine how manipulation of specific ILC subtypes affects both the immune and tissue landscapes. Overall, the clinical implications of ILC-directed therapies are immense. While regulating beneficial as opposed to pathologic responses of ILCs is no small feat, there is little doubt that successful manipulation of ILCs will have an exceptional effect on a multitude of human diseases.

## Figures and Tables

**Table 1:**
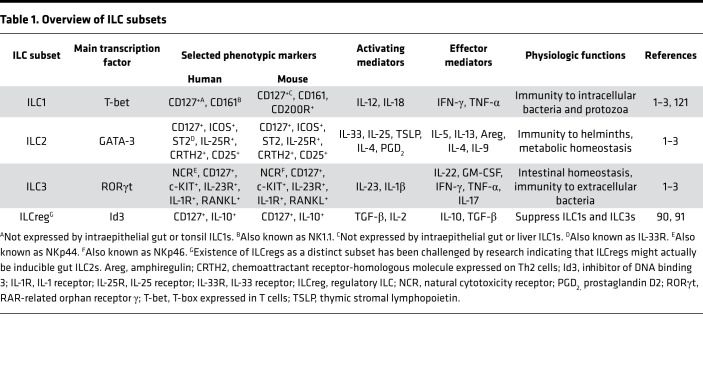
Overview of ILC subsets

**Table 2:**
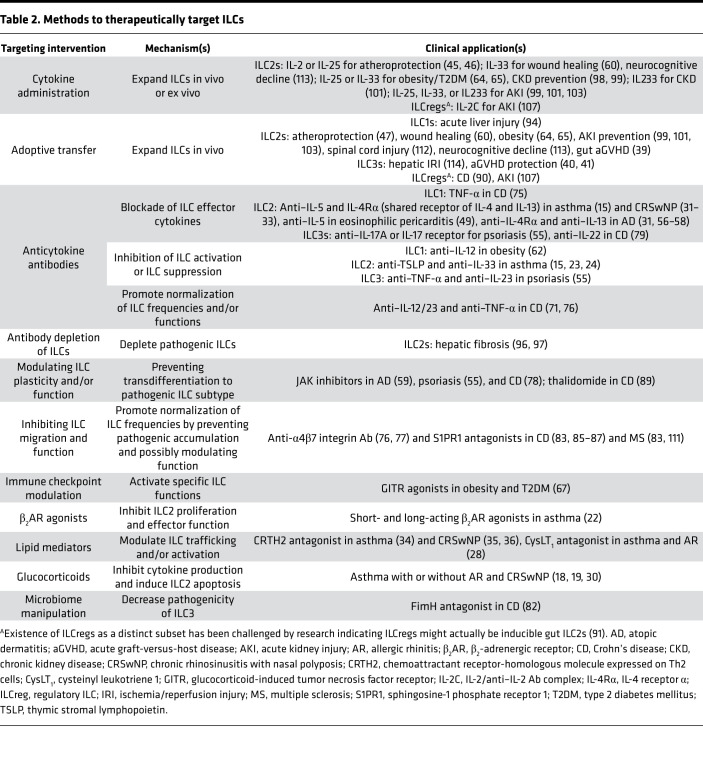
Methods to therapeutically target ILCs

## Version 1 03/22/2021

Electronic publication
